# The Influence of Personalised Sarcoma Care (PERSARC) Prediction Modelling on Clinical Decision Making in a Multidisciplinary Setting

**DOI:** 10.1155/2021/8851354

**Published:** 2021-10-21

**Authors:** H. S. Femke Hagenmaier, Annelies G. K. van Beeck, Rick L. Haas, Veroniek M. van Praag, Leti van Bodegom-Vos, Jos A. van der Hage, Stijn Krol, Frank M. Speetjens, Arjen H. G. Cleven, Ana Navas, Herman M. Kroon, Rieneke G. Moeri-Schimmel, Nicolette A. C. Leyerzapf, Michiel A. J. van de Sande

**Affiliations:** ^1^Department of Orthopaedic Surgery, Sint Maartenskliniek, Hengstdal 3, 6574 NA Ubbergen, Netherlands; ^2^Department of Orthopaedic Surgery, Leiden University Medical Centre, Albinusdreef 2, 2333 ZA Leiden, Netherlands; ^3^Department of Orthopaedic Surgery and Traumatology, University Hospital Antwerp, Wilrijkstraat 10, 2650 Edegem, Belgium; ^4^Department of Radiotherapy, Leiden University Medical Centre, Albinusdreef 2, 2333 ZA Leiden, Netherlands; ^5^Department of Radiotherapy, The Netherlands Cancer Institute, Plesmanlaan 121, 1066 CX Amsterdam, Netherlands; ^6^Department of Biomedical Data Sciences, Leiden University Medical Centre, Albinusdreef 2, 2333 ZA Leiden, Netherlands; ^7^Department of Surgery, Leiden University Medical Centre, Albinusdreef 2, 2333 ZA Leiden, Netherlands; ^8^Department of Clinical Oncology, Leiden University Medical Centre, Albinusdreef 2, 2333 ZA Leiden, Netherlands; ^9^Department of Pathology, Leiden University Medical Centre, Albinusdreef 2, 2333 ZA Leiden, Netherlands; ^10^Department of Radiology, Leiden University Medical Centre, Albinusdreef 2, 2333 ZA Leiden, Netherlands

## Abstract

**Background:**

With soft-tissue sarcoma of the extremity (ESTS) representing a heterogenous group of tumors, management decisions are often made in multidisciplinary team (MDT) meetings. To optimize outcome, nomograms are more commonly used to guide individualized treatment decision making.

**Purpose:**

To evaluate the influence of Personalised Sarcoma Care (PERSARC) on treatment decisions for patients with high-grade ESTS and the ability of the MDT to accurately predict overall survival (OS) and local recurrence (LR) rates.

**Methods:**

Two consecutive meetings were organised. During the first meeting, 36 cases were presented to the MDT. OS and LR rates without the use of PERSARC were estimated by consensus and preferred treatment was recorded for each case. During the second meeting, OS/LR rates calculated with PERSARC were presented to the MDT. Differences between estimated OS/LR rates and PERSARC OS/LR rates were calculated. Variations in preferred treatment protocols were noted.

**Results:**

The MDT underestimated OS when compared to PERSARC in 48.4% of cases. LR rates were overestimated in 41.9% of cases. With the use of PERSARC, the proposed treatment changed for 24 cases.

**Conclusion:**

PERSARC aids the MDT to optimize individualized predicted OS and LR rates, hereby guiding patient-centered care and shared decision making.

## 1. Introduction

Over the last decades, prognostic modelling has found its way into prediction of survival and possible adverse events in sarcoma care. Among the first of those nomograms were the Memorial Sloan Kettering Cancer Center (MSKCC) Sarcoma Nomogram [1], SIN-system [2], and Sarculator [3]. The implementation of these prediction models was mostly restricted to individual patient and physician use, but has found its way into risk assessment and (possibly) risk-based management for this patient group. For example, high-risk patients identified by Sarculator were thought to benefit more from an intensified treatment setting including chemotherapy in high-grade soft-tissue sarcoma [4, 5].

In 2017, a prognostic model was developed to predict the cumulative incidence of overall survival (OS) and local recurrence (LR) rates for patients with primary high-grade Extremity Soft-Tissue Sarcoma (ESTS) [6]. This model, Personalised Sarcoma Care (PERSARC), was developed to support shared decision making between patients and physicians by providing better insight into individualised OS and LR estimates for different treatment options. PERSARC was subsequently made publicly available as a mobile application through app stores and the website of the Leiden University Medical Centre (LUMC), for both healthcare professionals and patients. Up to now, it has been used as an informative tool for general information only, as the implications of the use of PERSARC in clinical practice and its effect on shared decision making have yet to be established.

Due to the heterogeneity of ESTS [7], protocols for treatment decisions are not readily available or have not yet reached a broad consensus. To optimize patient outcome, management decisions are often formulated in multidisciplinary team (MDT) meetings which are still regarded the standard of care. Therefore, some treatment decisions are, to some extent, based on the conclusions of case discussions and collective experience of the MDT.

This study was conducted to evaluate the influence of PERSARC prediction on final treatment advice in everyday clinical practice, such as in an MDT. Furthermore, the ability of the specialised sarcoma healthcare professionals to accurately predict OS and LR rates for different treatment scenarios in patients with ESTS was determined.

## 2. Materials and Methods

### 2.1. Study Design

To establish the added value of PERSARC when implemented in clinical practice, two MDTs were organised. During each meeting, treatment options and their supposed consequential OS and LR rates were estimated by dedicated sarcoma healthcare professionals in consensus. For both MDTs, at a minimum, the same radiation oncologist, sarcoma surgeon, oncological orthopaedic surgeon, medical oncologist, radiologist, radiation oncologist, and a pathologist participated.

In the first MDT, a comprehensive case presentation was given, including information about age, gender, clinical symptoms, medical history, and results of physical examination. Furthermore, radiological imaging and the histological diagnosis were reviewed. Based on these data, a treatment proposal was formulated by the MDT, and the expected 5-year OS and LR with this treatment plan were estimated for each case without the use of PERSARC. Three months later, a second meeting was organised for the identical group of sarcoma specialists, presenting the same anonymised and randomized cases. The only additional information provided to the MDT was the optional treatment modalities with accompanying predictions of OS and LR rates calculated by PERSARC. Thereafter, once again, consensus was reached on a treatment advice, using the PERSARC estimates for OS and LR.

### 2.2. Study Population

All cases were selected from the Leiden University Medical Centre (LUMC) Sarcoma Registry. Patients eligible for review with PERSARC had a high-grade (Fédération Nationale des Centres de Lutte Contre le Cancer (FNCLCC) grade III) primary ESTS with a minimum follow-up of 1 year. Patients presenting with local recurrence and/or distant metastasis at initial diagnosis were excluded from this study. The results of magnetic resonance imaging (MRI) and additional imaging were discussed by the musculoskeletal (MSK) oncology radiologist and histology by a dedicated sarcoma pathologist.

### 2.3. PERSARC

Due to the heterogeneity of ESTS with more than 60 subtypes, PERSARC has grouped some subtypes together. The ESTS subtypes included were all high-grade (FNCLCC grades II and III): angiosarcoma, malignant peripheral nerve sheath tumor, synovial sarcoma, spindle cell sarcoma, myxofibrosarcoma, liposarcoma, leiomyosarcoma, malignant fibrous histiocytoma/undifferentiated pleomorphic sarcoma (pleomorphic), soft-tissue sarcomas not otherwise specified, and other. The updated PERSARC application (Version 2.0) was used being readily available through the website of the LUMC (https://www.lumc.nl/org/oncologie-centrum/patienten/ziektes-en-aandoeningen/wekedelentumor/persarc/). Patient characteristics needed for the PERSARC prediction model were age, sex, sarcoma size in centimetres, tumor depth, histological type, and histological grade of the tumor. PERSARC prediction modelling of OS and LR for different treatment modalities was updated and externally validated in 2021, using 3826 ESTS patients treated with curative intend. Patients were added to the model development cohort, and grade was included in the model. External validation was performed with data from 1111 patients treated at a single tertiary centre [7].

### 2.4. Outcomes

The following possible treatment scenarios were formulated, as presented in Table 1.

During the first MDT, OS and LR rates without the use of PERSARC were estimated by the MDT for the preferred treatment modality for each case. During the second MDT, OS and LR rates were calculated with PERSARC for all the aforementioned treatment regimens and presented to the MDT on screen. The difference between estimated OS/LR rates and PERSARC OS/LR rates was calculated (∆OS and ∆LR), given as a positive or negative percentage to determine the degree of over- or underestimation of OS and LR rates by the MDT. All MDT estimations within 5% of PERSARC predictions were considered normal variance. Furthermore, the chosen treatment proposals of the first and the second multidisciplinary sarcoma team meeting were compared for each patient. Variations in preferred treatment regimens were noted.

### 2.5. Analysis

All statistical analyses were conducted with SPSS 20.0 (IBM SPSS Statistics for Windows, Version 20.0. Armonk, NY: IBM Corp). The Shapiro–Wilk test was used to test whether variables were normally distributed. In normally distributed data, mean and standard deviation were given. In nonnormally distributed data, median and range were used. A *p* value of 0.05 was defined as statistically significant.

## 3. Results

### 3.1. Study Population

Thirty-six patients that met the study criteria were randomly chosen from the LUMC Sarcoma Registry and included in this study. There were 19 males and 17 females included for review, and mean age at diagnosis was 55.9 years. All patients presented with a high-grade ESTS without distant metastasis. The mean tumour size at presentation was 9.5 cm (±5.9). Thirteen patients presented with a liposarcoma, 6 of which had a myxoid liposarcoma. All demographic data are presented in Table 2.

### 3.2. Overall Survival and Local Recurrence

Table 3 summarizes the treatments determined for each case and the accompanying estimated OS and LR rates without and with the use of PERSARC. The MDT proposed neoadjuvant radiotherapy followed by an intended R0 resection for 10 patients (27.7%) and neoadjuvant radiotherapy followed by an expected R1 resection for 13 patients (36.1%). In 2 cases (5.6%), neoadjuvant radiotherapy followed by an R2 resection was expected based on patient and tumor characteristics. The MDT proposed surgery as monotherapy in 7 patients; in 2 cases (5.6%), an expected R0 resection was performed, and in 5 patients (13.8%), an amputation was advised. However, since amputation cannot yet be entered in the PERSARC app as a treatment option and, therefore, no reliable PERSARC prediction for OS/LR could be made, these data have been removed from this analysis.

**Figure 1:**
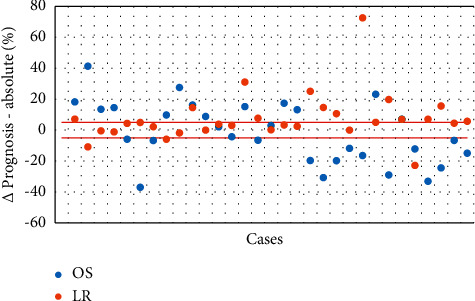
Differences in the prognosis of OS/LR.

Figure 1 shows the ∆OS and ∆LR for each individual case, expressed in percentages. A 5% positive or negative deviation from the absolute difference between PERSARC and the MDT was considered to be within normal range.

Regarding overall survival, the MDT underestimated OS when compared to PERSARC in 48.4% of cases (15/31). They overestimated OS in 41.9% (13/31) and predicted OS correctly in 9.7% of cases (3/31). For local recurrence, the MDT were more successful in their predictions, correctly estimating LR in 48.4% of our study population (15/31). It overestimated LR in 41.9% (13/31) and underestimated LR in 9.7% of cases (3/31).

### 3.3. PERSARC

Table 4 summarizes the proposed treatment regimens determined in the first meeting without the use of PERSARC and the second meeting using PERSARC. With the use of PERSARC, the proposed treatment changed in 24 cases (66.7%). In 25 instead of 10 patients, the preferred treatment protocol was neoadjuvant RT and an intended R0 resection (69.4% versus 27.7%, respectively). Six additional patients would receive neoadjuvant RT and an expected R0 or R1 resection instead of surgery only.

In the surgery-only group, with the use of PERSARC, 7 additional patients were advised to receive an intended R0 instead of an expected R1 resection (16.7% vs. 36.1%, respectively). No intralesional resections were advised. Furthermore, there was a preference for amputation in only 1 patient (2.8%), unlike 5 (13.8%) without the use of PERSARC. With PERSARC, the MDT decided that there was no indication for adjuvant radiotherapy in patients that were regarded in need for radiotherapy in the first MDT without the use of PERSARC.

Table 5 further elaborates on the changes in the treatment protocol that were made with the use of PERSARC on a case-by-case basis.

## 4. Discussion

In this study designed to clinically evaluate the use of the PERSARC prediction model, we found that the use of PERSARC caused a variation in the preferred treatment option in 66.7% of cases. Furthermore, dedicated sarcoma specialists were better able to accurately predict local recurrence than overall survival (9.7% versus 48.4%, respectively) in a multidisciplinary setting. Overall survival was underestimated by the MDT in 48.4%, compared to 9.7% underestimation for local recurrence. Although they predicted local recurrence accurately more often, the MDT still overestimated LR in the remainder (41.9%) of our study population.

Due to its heterogeneity and complex nature with a diverse clinical behavior depending on subtype, several previous studies have emphasized the importance of multidisciplinary care and dedicated team meetings to optimize outcome in the treatment of ESTS [8–11]. Although some have tried to address the quality and influence of MDT's on clinical decision making, none have assessed the predictive abilities of the multidisciplinary team and its members regarding the estimation of LR and OS rates in ESTS patients. In the current study, we found a slight overestimation of the local recurrence rate by the MDT (in 41.9%), which may have led to a collective underestimation of overall survival. As a result, the MDT may have chosen a more individual patient-care-based approach in some patients, instead of a more aggressive approach with curative intent. The use of the PERSARC prediction model caused a change in preferred treatment modality in 24 patients (66.7% of cases), mainly based on predicted higher overall survival rates by PERSARC. Therefore, PERSARC helps clinicians to estimate a more realistic prognosis of expected recurrence rates and life expectancy. This can potentially lead to a more frequent choice for limb salvage treatment, while maintaining comparable survival rates for these individual patients.

Looking at the different suggested treatment options, surgery with neoadjuvant radiotherapy was the treatment regimen most frequently preferred. Postoperative radiotherapy was completely discarded by the MDT as a potential treatment option (1 patient treated with adjuvant radiotherapy without PERSARC and none with PERSARC). Although the literature reports a poorer long-term functional outcome of the affected extremity for patients treated with postoperative radiotherapy, mainly due to the higher postoperative radiation dose, wound complications are much more common after preoperative radiotherapy [12–15]. As these short-term complications usually are manageable, they may be recognized as more acceptable, with better understanding of the predicted survival and local recurrence rates.

Even though PERSARC provides a validated prediction of LR and OS rates, there are some limitations that have to be considered [6]. First, the study population consisted of a relatively small number of patients, selected from the LUMC Sarcoma Registry, based on retrospective statistics. Second, the influence of comorbidity on overall survival is a well-known prognostic factor [16], which has not yet been implemented in the PERSARC model. Although the dedicated sarcoma team was provided with data about clinical condition and known comorbidities, the influence on treatment effectiveness, overall survival, and expected quality of life still needs to be considered in the final (shared) decision making.

We conclude that this study illustrates the additional individualized value of the PERSARC prediction model in clinical decision making. PERSARC provides specialised medical sarcoma professionals with improved insight in predicted local recurrence rates and overall survival chances for ESTS patients regarding different treatment modalities. In this respect, it may prove to be a valuable tool toward patient-centered care and shared decision making.

## Figures and Tables

**Table 1:** Treatment scenarios.

Neoadjuvant radiotherapy	Surgery only	Adjuvant radiotherapy
+Surgery R0	R0	+Surgery R0
+Surgery R1	R1	+Surgery R1
+Surgery R2	R2	+Surgery R2
	Amputation	

**Table 2:** Patient demographics.

	*N* (%)
Total	36
Age at diagnosis (mean (SD))	55.9 (21.2)

*Gender (%)*	
Male	19 (53.0%)
Female	17 (47.0%)

*Depth (%)* ^*+*^	
Superficial	13 (36.0%)
Deep	23 (64.0%)

Size in cm (mean (SD))	9.5 (±5.9)

*Histology (%)*	
Myxofibrosarcoma	4 (11.1%)
MPNST	4 (11.1%)
Synovial sarcoma	3 (8.3%)
MFH/UPS	2 (5.6%)
Liposarcoma	13 (36.1%)
Other	10 (27.8%)

^+^Depth: relative to the investing fascia; N: number of patients; MFH/UPS: malignant fibrous histiocytoma/undifferentiated pleomorphic sarcoma; MPNST: malignant peripheral nerve sheath tumor.

**Table 3:** Estimated and calculated OS and LR for each case with and without PERSARC.

Case	Treatment proposal	OS/LR MDT estimation (%)		OS/LR PERSARC prognosis (%)		∆ prognosis (∆—% absolute)	
		OS	LR	OS	LR	∆OS	∆LR
1	R1	92.5	30.0	74.2	22.8	18.3	7.2
4	Neoadjuvant RT, R1	82.5	5.0	41.1	15.8	41.4	−10.8
6	Neoadjuvant RT, R1	27.5	20.0	14.0	20.4	13.5	−0.4
7	Neoadjuvant RT, R1	70.0	12.5	55.4	13.6	14.6	−1.1
8	R0	20.0	17.5	25.9	13.1	−5.9	4.4
9	Neoadjuvant RT, R1	40.0	15.0	77.0	10.0	−37.0	5.0
10	Neoadjuvant RT, R2	65.0	17.5	71.7	15.3	−6.7	2.2
13	Neoadjuvant RT, R1	82.5	5.0	72.7	10.9	9.8	−5.9
14	Neoadjuvant RT, R2	57.5	22.5	29.9	24.3	27.6	−1.8
15	Neoadjuvant RT, R0	65.0	17.5	81.3	2.9	−16.3	14.6
16	Neoadjuvant RT, R0	80.0	5.0	71.1	5.0	8.9	0.0
17	Neoadjuvant RT, R1	82.5	12.5	80.4	8.7	2.1	3.8
18	Neoadjuvant RT, R0	72.5	7.5	76.7	4.4	−4.2	3.1
19	R0	70.0	45.0	54.7	13.9	15.3	31.1
20	Neoadjuvant RT, R0	72.5	10.0	79.0	2.3	−6.5	7.7
21	Neoadjuvant RT, R0	80.0	5.0	77.0	4.8	3.0	0.2
22	Neoadjuvant RT, R0	75.0	10.0	57.6	6.6	17.4	3.4
23	Neoadjuvant RT, R1	62.5	12.5	49.2	10.0	13.3	2.5
24	R1, adjuvant RT	17.5	55.0	37.1	29.9	−19.6	25.1
25	Neoadjuvant RT, R1	40.0	22.5	70.7	7.8	−30.7	14.7
26	Neoadjuvant RT, R1	60.0	20.0	79.7	9.3	−19.7	10.7
27	Neoadjuvant RT, R1	60.0	10.0	71.8	10.0	−11.8	0.0
28	R2	12.5	100.0	28.9	27.3	−16.4	72.7
29	Neoadjuvant RT, R0	65.0	10.0	41.8	4.9	23.2	5.1
30	Neoadjuvant RT, R1	15.0	30.0	44.0	10.2	−29.0	19.8
31	Neoadjuvant RT, R0	85.0	10.0	77.9	3.0	7.1	7.0
32	R2	5.0	30.0	17.1	52.8	−12.1	−22.8
33	Neoadjuvant RT, R1	20.0	20.0	53.1	12.9	−33.1	7.1
34	Neoadjuvant RT, R1	35.0	25.0	59.4	9.4	−24.4	15.6
35	Neoadjuvant RT, R0	60.0	10.0	66.6	5.5	−6.6	4.5
36	Neoadjuvant RT, R0	65.0	10.0	79.9	4.3	−14.9	5.7

RT: radiotherapy.

**Table 4:** Chosen treatment options with and without the use of PERSARC.

	Without PERSARC, *N* (%)	With PERSARC, *N* (%)
*Neoadjuvant radiotherapy*		
Surgery R0	10 (27.7%)	25 (69.4%)
Surgery R1	13 (36.1%)	6 (16.7%)
Surgery R2	2 (5.6%)	0 (0.0%)

*Surgery*		
Amputation	5 (13.8%)	1 (2.8%)
R0	2 (5.6%)	1 (2.8%)
R1	1 (2.8%)	3 (8.3%)
R2	2 (5.6%)	0 (0.0%)

*Adjuvant radiotherapy*		
Surgery R0	0 (0.0%)	0 (0.0%)
Surgery R1	1 (2.8%)	0 (0.0%)
Surgery R2	0 (0.0%)	0 (0.0%)

**Table 5:** Changes in the treatment protocol per case (*N* = 24) with the use of PERSARC.

Case	Without PERSARC	With PERSARC
3	Amputation	Neoadjuvant RT, R1
4	Neoadjuvant RT, R1	Neoadjuvant RT, R0
5	Amputation	Neoadjuvant RT, R0
6	Neoadjuvant RT, R1	Neoadjuvant RT, R0
7	Neoadjuvant RT, R1	Neoadjuvant RT, R0
8	R0	Neoadjuvant RT, R0
9	Neoadjuvant RT, R1	Neoadjuvant RT, R0
10	Neoadjuvant RT, R2	Neoadjuvant RT, R1
11	Amputation	Neoadjuvant RT, R0
12	Amputation	Neoadjuvant RT, R1
13	Neoadjuvant RT, R1	Neoadjuvant RT, R0
14	Neoadjuvant RT, R2	Neoadjuvant RT, R1
19	R0	R1
23	Neoadjuvant RT, R1	Neoadjuvant RT, R0
24	R1, adjuvant RT	R0
25	Neoadjuvant RT, R1	Neoadjuvant RT, R0
26	Neoadjuvant RT, R1	Neoadjuvant RT, R0
27	Neoadjuvant RT, R1	Neoadjuvant RT, R0
28	Neoadjuvant RT, R1	Neoadjuvant RT, R0
30	Neoadjuvant RT, R1	R1
31	Neoadjuvant RT, R0	Neoadjuvant RT, R1
32	R2	Neoadjuvant RT, R0
33	Neoadjuvant RT, R1	Neoadjuvant RT, R0
34	Neoadjuvant RT, R1	Neoadjuvant RT, R0

RT: radiotherapy.

## Data Availability

There are no supporting data available for the current manuscript.
